# The role of glycemic control and symptoms and symptom clusters in breast cancer survivors with type 2 diabetes

**DOI:** 10.1007/s00520-025-09434-5

**Published:** 2025-04-10

**Authors:** Susan Storey, Xiao Luo, Susan Ofner, Susan M. Perkins, Diane Von Ah

**Affiliations:** 1 Indiana University School of Nursing, Indianapolis, IN 46260 USA; 2 Department of Management Science and Information Systems, School of Business, Oklahoma State University, Stillwater, OK USA; 3 Department of Biostatistics and Health Data Science, School of Medicine and Richard M. Fairbanks School of Public Health, Indiana University, 410 W 10 th Street, Suite 3000, Indianapolis, IN 46202 USA; 4 Cancer Research, Center for Healthy Aging, Self-Management and Complex Care, College of Nursing, The Ohio State University (OSU), 394 Newton Hall, 1585 Neil Avenue, Columbus, OH 43210 USA; 5 Comprehensive Cancer Center, Cancer Control Program, OSU, 394 Newton Hall, 1585 Neil Avenue, Columbus, OH 43210 USA

**Keywords:** BCS, Glycemic control, Type 2 diabetes

## Abstract

**Purpose:**

The purpose of the study was to describe the type and number of symptoms and examine symptom clusters of breast cancer survivors (BCS) with diabetes (type 2) by glycemic control (HbA1c < 7 or ≥ 7%).

**Methods:**

A retrospective cohort study was conducted. Symptom data were extracted from clinical notes in the electronic health record. BCS (stage I-III) diagnosed between 2007 and 2019 had diabetes, and at least one HbA1c within 8 months of initial chemotherapy was included. Zero-inflated negative binomial regression analysis was used to examine total symptoms by glycemic control. Exploratory factor analysis was conducted to identify symptom clusters.

**Results:**

Three hundred twenty-seven BCS met the inclusion criteria. Two symptom clusters were identified in BCS with HbA1c ≥ 7%: a psychoneurological cluster (anxiety, fatigue, peripheral neuropathy, and depression) and a gastrointestinal cluster (vomiting, nausea, and constipation). Two symptom clusters were identified in BCS with HbA1c < 7% a mixed gastrointestinal/psychoneurological cluster (vomiting, nausea, peripheral neuropathy, fatigue, and constipation) and a mental health symptom cluster (depression and anxiety).

**Conclusion:**

The symptom clusters of BCS differed by glycemic control. Prospective research studies are needed to examine the role of glycemic control in symptoms in BCS with diabetes. Understanding the influence of glycemic control can help providers identify BCS at high risk for troublesome symptoms and symptom clusters, thereby facilitating interventions that target glycemic control, potentially mitigating symptoms, and symptom clusters, and improving outcomes for BCS with diabetes.

**Supplementary Information:**

The online version contains supplementary material available at 10.1007/s00520-025-09434-5.

## Introduction

Diabetes (type 2) has been shown to predict worse outcomes (e.g., disease progression, decreased survival) among breast cancer survivors (BCS) [1–3]. Recent findings suggest that poor glycemic control may play a major role in these poor outcomes [4, 5]. Poor glycemic control, particularly hyperglycemia, a common characteristic of diabetes, is estimated to occur in up to 30% of the over 38 million BCS in the USA [6, 7]. Poor glycemic control is frequently exacerbated by the cancer diagnosis, the treatment of cancer and concurrent metabolic changes, all of which have been associated with poorer health-related outcomes in cancer survivors [5, 8–11]. Specifically, hyperglycemia has been associated with a reduction in the effectiveness of cancer treatment through increased resistance to chemotherapy agents and decreased apoptosis of cancer cells, facilitating the escape of cancer cells leading to cancer progression [12–15].

BCS and people with diabetes experience similar symptoms that may co-occur known as symptom clusters [16–19]. Common shared symptoms include anxiety, cognitive problems, peripheral neuropathy, diarrhea, constipation, vomiting, nausea, fatigue, physical function problems, sleep problems, and depression [16, 18]. Only a few studies have examined the symptoms of BCS with diabetes. Among those studies, researchers noted diabetes was associated with cancer-related fatigue, cognitive impairment, and a risk factor for peripheral neuropathy in BCS [17, 20, 21]. However, these studies were retrospective or assessed individual symptoms and did not include a comprehensive examination of the symptoms or symptom clusters of BCS with comorbid diabetes, nor did they examine the influence of glycemic control on symptoms or symptom clusters.

Poor glycemic control, specifically hyperglycemia, increases oxidative stress and inflammatory responses, which may cause and/or intensify symptoms/symptom clusters [8]. Previous studies examining glycemic control in BCS have primarily focused on health-related outcomes such as survival, mortality, progression, recurrence, infection, and resource utilization [13, 14, 22]. Thus, there is a paucity of research on the role of glycemic control on the symptoms and symptom clusters of BCS with diabetes. Glycemic control is a modifiable risk factor that, if addressed and managed, may alleviate symptoms and symptom clusters.

This study examined the role of glycemic control, using glycated hemoglobin (HbA1c), on the symptoms and symptom clusters of BCS with diabetes following the initiation of chemotherapy. Specific aims were to (1) describe the type and number of symptoms in BCS by glycemic control (HbA1c < 7% or ≥ 7%) and (2) examine the type and number of symptoms and symptom clusters by glycemic control. Findings from this study will assist in improving our understanding of the role of glycemic control and identify potential avenues for interventional research to mitigate symptoms and symptom clusters in BCS with diabetes.

## Methods

### Study design and sample

This pilot study was a retrospective cross-sectional cohort study. Electronic health records (EHR) from a large statewide health repository were used to extract data. The repository, managed by the Regenstrief Institute Data Core, includes clinical information from all affiliated health systems in Indiana, USA [23]. This study was performed in line with the principles of the Declaration of Helsinki. Approval was granted by the Indiana University Institutional Review Board (Date February 19, 2021, No. 10526).

Eligibility criteria included a diagnosis of breast cancer (stage I-III disease), at least one HbA1c test within 8 months following initiation of chemotherapy which is during the acute treatment period, and a diagnosis of diabetes per International Classification of Disease (ICD) codes, between 2007 and 2019. This time period was chosen because it was pre-pandemic, and the symptom data was not confounded by COVID- 19 or long COVID symptoms.

### Data extraction

Demographic and medical characteristics were extracted from the EHR for the 8-month period after the date of the initial chemotherapy treatment, which spans the active treatment period and the immediate post-treatment period when symptoms are potentially at their highest. These characteristics included age, race, cancer stage, BMI, comorbidities, anemia, steroid administration, and HbA1c laboratory values, all of which are associated with increased risk for breast cancer, diabetes, and/or poor glycemic control [24–26]. A modified Charlson Comorbidity Index score (eliminating cancer and diabetes) was used to account for comorbid conditions. Higher scores indicate a higher disease burden [27]. The presence of anemia (ICD 9/10) and steroid administration (pharmacy data) were collected and coded as dichotomous variables (yes/no). HbA1c reflects the average blood glucose for the previous 120 days and is the standard measurement used to assess glycemic control [28]. For this study, adequate glycemic control was defined as an HbA1c of < 7%, and poor glycemic control was defined as an HbA1c ≥ 7% per the ADA guidelines [28].

The computational methods used to extract symptoms from the clinical notes of the EHR are described elsewhere [29]. Briefly, we developed natural language algorithms to identify common phrases or words in the clinical notes that matched the symptoms of interest. We identified a priori 11 of the most reported symptoms (anxiety, cognitive problems, peripheral neuropathy, diarrhea, constipation, vomiting, nausea, fatigue, physical function problems, sleep problems, and depression) among cancer survivors and people with diabetes [16–18]. Biomedical language embedding techniques were used to identify the synonyms for the symptoms [30]. For example, for sleep problems, synonyms included “can’t fall asleep,” “can’t stay asleep,” “restless sleep,” “nightmares,” “can’t sleep,” “insomnia,” “restlessness,” “interrupted sleep,” and “sleeplessness” [31]. The presence of symptoms was denoted as yes or no. If no clinical notes were found, no symptoms were coded. The frequency of each symptom within the time frame was calculated. See Supplement 1 for the full list of symptom synonyms.

### Data analysis

Demographics and comorbidities were compared between those with and without HbA1c lab tests by means of Wilcoxon rank sum and chi-square tests. Using these same methods, characteristics between BCS with adequate (HbA1c < 7%) and poor (HbA1c ≥ 7%) glycemic control were compared.

For the primary aim to describe the type and number of symptoms by glycemic control, chi-square tests were used to compare occurrences of specific symptoms by glycemic control (HbA1c < 7% versus HbA1c ≥ 7%). Histograms of the number of symptoms for each group revealed distributions that were zero-inflated and right-skewed. Therefore, a zero-inflated negative binomial (ZINB) model was used, which simultaneously modeled the probability of having no symptoms and the total number of symptoms. Bivariate models with independent variables of glycemic control (HbA1c), age, race, cancer stage, modified Charlson score, anemia, and steroid use were conducted. Multiple regression models were run and included all characteristics. Variables with small cell counts (steroids, white race, anemia, modified Charlson) were removed, resulting in a final model that included HbA1c ≥ 7%, age, and cancer stage.

For the secondary aim to examine the number and type of symptom clusters by glycemic control, individual symptom occurrence (yes/no) was assessed using exploratory factor analysis (principal axis factoring with promax rotation) by glycemic control (HbA1c < 7% versus HbA1c ≥ 7%). Because few BCS had the following symptoms—cognition, diarrhea, physical function, or sleep—recorded in the EHR, these symptoms were omitted from the factor analysis. Symptoms with loadings of 0.4 or higher were considered correlated and a symptom cluster [32]. All analyses were conducted using SAS Software version 9.4 (Copyright © 2016; SAS Institute Inc., Cary, NC, USA).

## Results

### Demographic and medical characteristics

A total of 2456 BCS charts were reviewed; of these, 327 (13.3%) had at least one HbA1c lab test within the 8 months following the initial chemotherapy and were included in the analysis. BCS with an HbA1c lab test were significantly more likely to be of non-white race; have cardiovascular disease, pulmonary disease, mild liver disease, renal disease, and rheumatic disease; and have higher modified Charlson scores than those without HbA1c lab tests. Of the 327 BCS with diabetes, 148 (45.3%) had an HBA1c ≥ 7% on their first lab value post-chemotherapy. In addition, those with HbA1c ≥ 7% were significantly more likely to have used steroids and had mild liver disease and less likely to have chronic pulmonary disease than BCS with HbA1c < 7% (Table 1).

**Table 1:** Demographic and medical characteristics of breast cancer survivors with diabetes by glycemic control (*n* = 327)

Characteristic		HbA1c ≥ 7%, *n* = 148 (45.3%)	HbA1c < 7%, *n* = 179 (54.7%)	*p*-value
Age at cancer diagnosis	Mean ± SD	58.6 ± 10.3	60.5 ± 9.9	0.0784
	Median (Min, Max)	59.0 (37.0, 80.0)	61.0 (31.0, 82.0)	
Race	White	107 (72.3)	144 (80.4)	0.0824
	Non-White	41 (27.7)	35 (19.6)	
Spanish or Hispanic origin	Non-Spanish/non-Hispanic	145 (98.0)	172 (96.1)	0.4176
	Mexican (includes Chicano)	0 (0.0)	1 (0.6)	
	Puerto Rican	2 (1.4)	0 (0.0)	
	Other specified Spanish/Hispanic origin	0 (0.0)	1 (0.6)	
	Spanish, + NOS/Hispanic, NOS/Latino, NOS	1 (0.7)	2 (1.1)	
	Spanish surname only	0 (0.0)	2 (1.1)	
	Unknown whether Spanish or not	0 (0.0)	1 (0.6)	
First BMI after chemotherapy start	N	19	12	0.5565
	Mean ± SD	40.5 ± 25.3	37.4 ± 8.4	
	Median (Min, Max)	32.1 (23.8, 137.0)	37.7 (26.0, 52.0)	
Cancer stage	1	42 (28.4)	54 (30.2)	0.9391
	2	68 (45.9)	80 (44.7)	
	3	38 (25.7)	45 (25.1)	
Anemia	No	131 (88.5)	153 (85.5)	0.4183
	Yes	17 (11.5)	26 (14.5)	
Steroid use	No	132 (89.2)	173 (96.6)	0.0074
	Yes	16 (10.8)	6 (3.4)	
Charlson: AIDS	No	148 (100.0)	179 (100.0)	
Charlson: cardiovascular disease (cerebrovascular disease, congestive heart failure, myocardial infarction)	No	128 (86.5)	148 (82.7)	0.3452
	Yes	20 (13.5)	31 (17.3)	
Charlson: chronic pulmonary disease	No	129 (87.2)	135 (75.4)	0.0074
	Yes	19 (12.8)	44 (24.6)	
Charlson: dementia	No	148 (100.0)	179 (100.0)	
Charlson: hemiplegia or paraplegia	No	148 (100.0)	177 (98.9)	0.5030
	Yes	0 (0.0)	2 (1.1)	
Charlson: mild liver disease	No	133 (89.9)	173 (96.6)	0.0128
	Yes	15 (10.1)	6 (3.4)	
Charlson: moderate or severe liver disease	No	148 (100.0)	178 (99.4)	0.9999
	Yes	0 (0.0)	1 (0.6)	
Charlson: peptic ulcer disease	No	148 (100.0)	177 (98.9)	0.5030
	Yes	0 (0.0)	2 (1.1)	
Charlson: renal disease	No	137 (92.6)	170 (95.0)	0.3664
	Yes	11 (7.4)	9 (5.0)	
Charlson: rheumatic disease	No	143 (96.6)	174 (97.2)	0.7597
	Yes	5 (3.4)	5 (2.8)	
Modified Charlson score*	0	97 (65.5)	110 (61.5)	0.6753
	1	28 (18.9)	35 (19.6)	
	2 +	23 (15.5)	34 (19.0)	

+ *NOS* not otherwise specified

^*^Charlson score calculated without inclusion of cancer or diabetes diagnoses

### Types of symptoms by glycemic control

Of the BCS with diabetes and an HbA1c test (*n* = 327), the majority 276 (84.4%) had at least one symptom. The most common symptoms included nausea (*n* = 210; 64.2%), vomiting (*n* = 194; 59.3%), fatigue (*n* = 184; 56.3%), anxiety (*n* = 118; 36.1%), constipation (*n* = 114; 34.9%), peripheral neuropathy (*n* = 109; 33.3%), depression (*n* = 109; 33.3%), sleep disturbance (*n* = 25; 7.6%), physical function problems (*n* = 18; 5.5%), and diarrhea (*n* = 16; 4.9%). No significant differences were noted in symptom occurrence by glycemic control (Table 2).

**Table 2:** Difference in symptom type and occurrence by glycemic control

Symptom	Overall, *n* = 327	HbA1c ≥ 7%, *n* = 148 (45.3%)	HbA1c < 7%, *n* = 179 (54.7%)	*p*-value^1^
Anxiety	118 (36.1)	56 (37.8)	62 (34.6)	0.5486
Cognitive	0 (0.0)	0 (0.0)	0 (0.0)	–-
Peripheral neuropathy	109 (33.3)	51 (34.5)	58 (32.4)	0.6945
Diarrhea	16 (4.9)	7 (4.7)	9 (5.0)	0.9010
Constipation	114 (34.9)	51 (34.5)	63 (35.2)	0.8894
Vomiting	194 (59.3)	94 (63.5)	100 (55.9)	0.1611
Nausea	210 (64.2)	97 (65.5)	113 (63.1)	0.6506
Physical function	18 (5.5)	9 (6.1)	9 (5.0)	0.6777
Sleep	25 (7.6)	13 (8.8)	12 (6.7)	0.4811
Fatigue	184 (56.3)	92 (62.2)	92 (51.4)	0.0508
Depression	109 (33.3)	49 (33.1)	60 (33.5)	0.9374

^1^*p*-value from chi-square test

### Number of symptoms by glycemic control

Table 3 presents results from the ZINB model of the number of symptoms as explained by glycemic control (HbA1c < 7% or ≥ 7%), controlling for age and cancer stage. In both models, glycemic control was not statistically significantly associated with the number of symptoms or the likelihood of having symptoms. However, fatigue was trending toward significance (*p* = 0.0508). Age and cancer stage were not statistically associated with the total number of symptoms or the likelihood of having symptoms.

**Table 3:** Total symptoms by glycemic control and prespecified covariates^1^

Variable		Measure	Statistic	95% confidence interval	*p*-value
HbA1c ≥ 7% only model (unadjusted results)					
HbA1c ≥ 7.0		IRR	1.21	(0.92, 1.60)	0.1670
		OR^2^	0.60	(0.03, 12.50)	0.7387
Multiple regression model (adjusted results)					
HbA1c ≥ 7.0		IRR	1.18	(0.90, 1.55)	0.2256
		OR^2^	0.98	(0.12, 8.33)	0.9871
Age at cancer diagnosis		IRR	1.00	(0.99, 1.02)	0.6588
		OR^2^	0.94	(0.85, 1.05)	0.3092
Stage	2	IRR	1.21	(0.87, 1.66)	0.2536
		OR^2^	4.35	(0.22, 100.00)	0.3349
	3	IRR	1.14	(0.78, 1.65)	0.4944
		OR^2^	1.85	(0.14, 25.00)	0.6379

Abbreviations: *IRR* incidence rate ratio, *OR* odds ratio

^1^Models for both the negative binomial and logistic parts included age + cancer stage + HbA1c ≥ 7.0. The negative binomial modeled the natural log of the number of total symptoms for a given lab test, given the total was not zero. The logistic model modeled the natural log of the odds of not having any symptoms for a given lab test

^2^OR presented are of having at least 1 symptom

### Symptom clusters by glycemic control

Factor analysis was conducted with the remaining seven symptoms (anxiety, depression, fatigue, peripheral neuropathy, vomiting, nausea, and constipation). Table 4 shows the factor loadings for each symptom. In BCS with HbA1c ≥ 7%, we identified two symptom clusters. Cluster one was a psychoneurological cluster consisting of anxiety, fatigue, peripheral neuropathy, and depression symptoms (highest to lowest factor loadings). Cluster two was a gastrointestinal cluster made up of vomiting, nausea, and constipation symptoms (highest to lowest factor loadings). The psychoneurological cluster explained 85% of the total variance between the occurrence of symptoms, and the gastrointestinal factor explained 15%.

**Table 4:** Factor analysis of symptom clusters by glycemic control

	(HbA1c ≥ 7.0%)		(HbA1c < 7.0%)	
	Factor 1	Factor 2	Factor 1	Factor 2
	Anxiety	Vomiting	Vomiting	Depression
Anxiety	0.810	0.014	0.186	0.589
Depression	0.529	0.195	− 0.051	1.022
Fatigue	0.748	0.099	0.555	0.029
Peripheral neuropathy	0.652	0.039	0.674	0.231
Vomiting	− 0.011	1.021	0.949	− 0.096
Nausea	0.054	0.829	0.920	0.013
Constipation	0.295	0.489	0.525	0.216
Percent of variance explained	85.9%	14.7%	78.7%	21.4%

Loadings with absolute value of 0.4 or greater are bolded

For BCS with HbA1c < 7%, we identified two symptom clusters. Cluster one was a mixed gastrointestinal/psychoneurological cluster consisting of vomiting, nausea, peripheral neuropathy, fatigue, and constipation symptoms (highest to lowest factor loadings). Cluster 2 was a mental health cluster consisting of depression and anxiety symptoms (highest to lowest factor loadings). The gastrointestinal/psychoneurological cluster explained 79% of the total variation of symptom occurrence, and the mental health cluster explained 21%.

## Discussion

To our knowledge, this is one of the first studies to use the novel approach of using HbA1c to examine the role of glycemic control in symptoms and symptom clusters among BCS with diabetes. Interestingly, in our study, only 13.3% of all BCS with diabetes during a critical treatment period had a documented HbA1c. Similarly, this finding was also noted by other researchers who reported glycemic monitoring of only 13% in a retrospective study of male survivors of genitourinary cancer (*n* = 30) [33]. The low frequency of HbA1c data in EHR during this critical period may represent the shift in healthcare providers’ focus from diabetes management to cancer treatment [34]. In a qualitative study of oncologists and primary care providers, they reported a lack of confidence in treating conditions outside of their specialty area and a lack of consistent communication between the specialties as barriers to care of cancer survivors with diabetes [35]. Adding to these challenges, cancer survivors with diabetes often report poorer adherence with diabetes management and medications [36]. It is plausible that challenges in diabetes management may perpetuate or exacerbate symptoms and symptom clusters in BCS. Collaboration between interdisciplinary providers from the onset of the cancer diagnosis is imperative to develop a plan of care that will ensure good glycemic control while BCS are receiving active treatment to improve both cancer and diabetes-related outcomes.

### Symptoms

The symptoms examined in our study (anxiety, depression, fatigue, peripheral neuropathy, vomiting, nausea, and constipation) align with the symptoms noted in other cross-sectional and longitudinal studies of BCS [26], which highlights the importance of assessing individual symptoms during acute treatment as well as throughout the cancer trajectory. In this study, we did not find a difference in the types of or number of symptoms of BCS with diabetes by glycemic control. We did note a trend toward a significance in fatigue, which is consistent with our previous work, where BCS with diabetes reported a higher symptom burden including more fatigue when compared to BCS without diabetes; however, that study did not examine the role of glycemic control on symptoms [17]. Poor glycemic control, specifically hyperglycemia, has been associated with a higher symptom burden among people with other cancer diagnoses [16, 37] and people with diabetes [38–40]. In people with diabetes and no cancer diagnosis, poor glycemic control has been associated with more physical and psychological symptoms [40–42]. The lack of group differences in the number of symptoms between those with adequate and poor glycemic control may be due to the limited number of pre-selected symptoms. In addition, other confounding factors such as routine steroid administration during cancer treatment and/or type of cancer treatment may have impacted symptom occurrence. Additionally, we did not use traditional standard symptom measurement tools; instead, we measured symptom presence (yes/no), with the number of reported symptoms being used to interpret symptom severity; thus, study findings are limited regarding severity.

There is a lack of studies examining the influence of glycemic control using HbA1c on symptoms and of cancer survivors with diabetes. We found only one study that examined the role of glycemic control using HbA1c on physical and psychological symptoms (fatigue, attentional function, pain, anxiety, depression, and sleep) in a sample of 244 cancer survivors (breast, gastrointestinal, gynecologic, and lung) [43].These researchers measured symptom severity, rather than the presence of symptoms, in cancer survivors and categorized them by no diabetes (HbA1c < 5.7%), pre-diabetes (HbA1c 5.7–6.4%), and diabetes (HbA1c ≥ 6.5%) [43]. The researchers failed to note an association between glycemic control and symptom severity; however, they postulated that it may be related to measuring glycemic control at a single time point versus measuring glycemic variability (hypoglycemia, normoglycemia, and hyperglycemia) over time, which may have a more deleterious influence on symptoms than hyperglycemia alone [43]. Prospective research studies are needed to examine the influence of glycemic control, including glycemic variability, on the presence, type, number, and severity of symptoms in BCS with diabetes.

It is plausible that BCS may have difficulty differentiating whether their symptoms are caused by cancer, its treatment, comorbid diabetes, and/or glycemic control. BCS and people with diabetes experience similar symptoms, some of which may be present prior to chemotherapy. Because of overlapping symptoms and potentially higher symptom burden at baseline, BCS with diabetes may report symptoms less frequently to their healthcare providers. Thorough assessment and monitoring of glycemic control in BCS with diabetes are imperative at baseline and throughout the treatment trajectory.

### Symptom clusters

Our previous work and that of others have demonstrated the deleterious impact of diabetes on symptom clusters in other cancer populations [44–48]; however, less is known about the influence of glycemic control on symptom clusters in BCS with diabetes. In this study, both groups of BCS with diabetes with HbA1c < 7% or ≥ 7% had two symptom clusters with minor variations in the individual symptoms that comprised the clusters. Specifically, we identified two symptom clusters among BCS with diabetes in both groups. Interestingly, we noted that BCS with diabetes and an HbA1c ≥ 7% had distinct psychoneurological (anxiety, fatigue, peripheral neuropathy, and depression) and gastrointestinal (vomiting, nausea, and constipation) symptom clusters. Psychoneurological symptoms are common in both BCS and people with diabetes and can worsen gastrointestinal symptoms [49–52]. It is possible the gastrointestinal symptom cluster we found in the BCS with diabetes and HbA1c > 7% group may be indicative of long-term poorer glycemic control (chronic hyperglycemia) which is known to cause gastroparesis and subsequent gastrointestinal symptoms [52, 53]. Whereas in BCS with diabetes and an HbA1c < 7%, we found a mixture of gastrointestinal/psychoneurological (vomiting, nausea, peripheral neuropathy, fatigue, and constipation) symptoms in one cluster and a distinct mental health cluster (depression and anxiety). The diagnoses of diabetes or breast cancer have independently been associated with anxiety and depression [54–56]. In our study, these symptoms were present regardless of glycemic control, suggesting mental health is an issue for BCS with multiple comorbid conditions. The influence of glycemic control on depression and anxiety of BCS has not been well documented. Emerging evidence from among people with diabetes has noted that long-term glycemic variability is associated with greater anxiety and depression [53, 57, 58]. More research is needed to examine the role of glycemic control on the symptoms and symptom clusters of BCS with diabetes.

The preliminary findings from our study demonstrate that glycemic control may play a role in the development of unique symptom clusters in BCS with diabetes. Previous research has highlighted the importance of identifying key symptoms that may trigger other symptoms to cluster together [47]. Future research to identify key symptoms and subsequent clusters is needed to develop targeted interventions which can reduce symptom burden for BCS with diabetes.

The symptom clusters we found in this study are similar to those noted previously in the BCS and diabetes literature [26, 48]. In a recent systematic review (*n* = 32 studies), So and colleagues noted the three most common symptom clusters among BCS during active treatment were a gastrointestinal cluster (nausea, lack of appetite), a pain-fatigue-sleep disturbance cluster, and a psychological cluster (anxiety-depression-worry-sadness-nervousness-irritability) [26]. While consistent with our findings, it is worth noting that the studies in that review did not include BCS with diabetes or examine glycemic control on the symptom clusters. The authors of that review noted that the utilization of different methodologies, symptom measures, and types of cancer treatment may influence symptom clusters and suggest future studies include homogenous groups of cancer survivors at consistent times during the treatment trajectory utilizing standardized measures to capture symptom clusters [26].

Longitudinal studies are warranted as they can capture changes in symptom clusters over time due to physiological changes associated with cancer diagnosis, treatment regimens, and glycemic control. Future studies using glycemic biomarkers like HbA1c to examine their role in symptom clusters can facilitate the identification of biological pathways that may contribute to symptom cluster formation and/or exacerbation in BCS with diabetes. Such studies can inform the development of effective patient-specific interventions tailored toward glycemic control and the potential mitigation of multiple symptom clusters.

### Strengths and limitations

Our study has several strengths. To date, research studies have focused on understanding the symptom cluster experience of breast cancer survivors in general, with a dearth representing those with comorbid diabetes. Our study contributes new knowledge regarding the role of glycemic control on the symptoms and symptom clusters of BCS with diabetes.

Experts recommend the use of innovative methods, such as utilizing large datasets for identifying symptom clusters within and across common chronic disease conditions [17, 19, 59]. The use of clinical notes provided real-time descriptions of symptoms and symptom clusters experienced by BCS with diabetes without the influence of predetermined symptom measurement tools. Moreover, using large datasets to examine symptom clusters in BCS with diabetes allowed us to observe differences in glycemic control between groups without imposing additional burdens on BCS with diabetes.

Our study had limitations. First, while innovative, using EHR has its limitations, particularly as it relates to clinical notes, which are typically used to support clinical practice rather than scientific research. It is possible that not all BCS report symptoms unless specifically asked about them or that if discussed with the healthcare provider, the conversation is not documented in the EHR. Additionally, it is feasible that some symptom expression terms were inadvertently omitted, thus influencing the symptoms and symptom clusters we found. However, we included broad symptom expression terms to allow us to capture more symptoms. It is imperative that standardized symptom measures be integrated into the EHR to facilitate treatment decisions made by healthcare providers, potentially reducing morbidity among high-risk populations such as BCS with diabetes.

Second, we used HbA1c values to determine glycemic control status. Our study yielded relatively few documented HbA1c lab results (13.3%), which limits the generalizability of our study. The lack of HbA1c lab tests in the EHR is an important finding and highlights the need for healthcare providers to be more diligent in incorporating these important measures into clinical practice to facilitate the best outcomes for BCS with diabetes. Although HbA1c is the standard measure of glycemic control, it may be affected by anemia caused by cancer and cancer treatment [28, 60]. The diagnosis of anemia in our study was low, which leads us to believe that this did not confound our findings. While HbA1c provides valuable information, it does not capture glycemic variations that commonly occur in people with diabetes. Recent research indicates that fluctuations in glycemic control may be more detrimental to the health-related outcomes and quality of life of cancer survivors than hyperglycemia alone [8, 43]. It is plausible that perturbations in glycemic control may further exacerbate symptoms and/or symptom clusters. Future studies should include additional glycemic measures such as fasting, random blood glucose, and/or continuous glucose monitoring while simultaneously measuring symptoms and symptom clusters.

Lastly, there were statistical limitations, including having few subjects with zero symptoms who were non-white race, used steroids, or had anemia, which limited our ability to include all the prespecified covariates in the logistic part of the ZINB model.

### Implications for practice

 As BCS are living longer, and the prevalence of diabetes is projected to continue to increase, healthcare providers will be challenged in the care of these comorbid conditions. Utilizing biomarkers like HbA1c to screen for glycemic control at baseline and throughout the cancer trajectory as standard of care will facilitate the identification of high risk BCS and allow for preemptive intervention, ongoing monitoring and treatment of hyperglycemia. Interdisciplinary collaboration among medical specialties in oncology and endocrinology is imperative to ensure optimal patient outcomes and to inform the development of guidelines for healthcare providers caring for this vulnerable population.

## Conclusion

This study is one of the first to examine the role of glycemic control (HbA1c) on the symptoms and symptom clusters in BCS with diabetes. We found BCS with HbA1c’s (> 7% and ≤ 7%) had distinct symptom clusters. Prospective research studies are warranted to validate these findings in real time and examine the role of glycemic control on the symptoms and symptom clusters in BCS with diabetes in real time. Understanding the influence of glycemic control can facilitate the development of interventions that improve glycemic control, potentially mitigating symptoms and symptom clusters and improving outcomes for BCS with diabetes.

## Supplementary Information

Below is the link to the electronic supplementary material.

Supplementary file1 (DOCX 16 KB)

## Data Availability

The datasets used and/or analyzed during the current study are available fro the corresponding author upon reasonable request.
