# Supplementary material for: The role of glycemic control and symptoms and symptom clusters in breast cancer survivors with type 2 diabetes
Source: Support Care Cancer. 2025 Apr 10;33(5):371. doi: 10.1007/s00520-025-09434-5 (PMC11985596; doi:10.1007/s00520-025-09434-5)
Supplement: Supplementary file 1 — Supplementary file1 (DOCX 16 KB) [file 520_2025_9434_MOESM1_ESM.docx]

**Supplement 1. Synonyms for Symptoms and Symptom Clusters in Breast Cancer Survivors**

| **Symptom Cluster** | **Symptoms and Synonyms** |
| --- | --- |
| **Anxiety** | anxiety, anxious, apprehensive, nervous, stressed, uptight, tensed, can’t relax, worry, worried, worrisome, panic, panicked, panic attack, irritable, overwhelmed, nervousness, fretful |
| **Cognitive issues** | cognitive issue, forgetful, chemo brain, fogginess, lack of concentration  difficulty remembering, memory loss, spaced out, can’t stay focused, can’t think straight, brain drain, loss of right words to say, forgetfulness, decreased concentration |
| **Peripheral Neuropathy** | peripheral neuropathy, numbness, tingling, burning, crawling, chest burning, leg burning, foot burning, eye numbness, hand numbness, upper extremity numbness, finger numbness, numbness of hand, facial numbness, numbness feet, leg numbness, numbness toe, tongue numbness, numbness of fingers, numbness extremity, thigh numbness, numb- ness thigh, numbness of extremities, numbness of upper arm, foot numbness, peripheral numbness, lip numbness, lower extremity numbness, feet numbness, extremity numbness, shoulder numb- ness, upper arm numbness, numbness tongue, numbness fingers, fingers numbness, numbness of finger, numbness foot, numbness lip, numbness of toe, neuropathic pain, peripheral neuropathic pain, limb numbness, toe numbness |
| **Gastrointestinal Issues** | anorexia, poor appetite, not hungry, no or decreased appetite, bad taste in mouth,  constipation, bloating, diarrhea, cramping, nausea, vomiting, thirsty, difficulty swallowing, bloating symptoms, bloated feeling, gas bloating, bloating gas, constipate, constipating, diarrhea, diarrhea symptoms, diarrheas, reduced appetite, decrease appetite, nausea/vomiting, morning vomiting, recurrent vomiting, acute vomiting, intermittent vomiting, vomiting symptoms, vomit, constipated, intractable vomiting, diarrhea vomiting, nauseated, persistent vomiting, acute constipation, chronic constipation, nausea emesis, nausea sickness, nauseas |
| **Physical Functions** | inability to care for self, can’t do the things I used to do, can’t keep up,  loss of stamina, unsteady gait, unsteady, falling, falls, gait changes, can’t walk, can’t walk far, shaky, slowing down, loss of strength, weak,  need help cooking/eating/driving/walking/bathing, |
| **Sleep issues** | can't sleep, insomnia, restlessness, wakeup, can’t fall asleep, can’t stay asleep, interrupted sleep, sleeplessness, can’t fall asleep, can’t stay asleep, restless sleep, nightmares |
| **Fatigue** | fatigue, listless, weary, weariness, lethargic, lethargy, no energy, tired, sleepy,  drowsy, exhausted, exhaustion, worn out, drained |
| **Depression** | depression, depressed, sad, unhappy, no appetite, failure to thrive, despair,  misery, melancholy, hopeless, down-hearted, despondent |
